# Resilient Hippocampal Gamma Rhythmogenesis and Parvalbumin-Expressing Interneuron Function Before and After Plaque Burden in *5xFAD* Alzheimer’s Disease Model

**DOI:** 10.3389/fnsyn.2022.857608

**Published:** 2022-05-11

**Authors:** Connie A. Mackenzie-Gray Scott, Kenneth A. Pelkey, Adam P. Caccavano, Daniel Abebe, Mandy Lai, Khayla N. Black, Nicolette D. Brown, Andrew J. Trevelyan, Chris J. McBain

**Affiliations:** ^1^Section on Cellular and Synaptic Physiology, NICHD - Eunice Kennedy Shriver National Institute of Child Health and Human Development, National Institutes of Health (NIH), Bethesda, MD, United States; ^2^Newcastle University Biosciences Institute, Newcastle University, Newcastle upon Tyne, United Kingdom

**Keywords:** gamma oscillations, *5xFAD*, parvalbumin interneurons, Alzheimer’s disease, carbachol, kainate

## Abstract

Recent studies have implicated impaired Parvalbumin Fast-Spiking Interneuron (PVIN) function as a precipitating factor underlying abnormalities in network synchrony, oscillatory rhythms, and cognition associated with Alzheimer’s disease (AD). However, a complete developmental investigation of potential gamma deficits, induced by commonly used carbachol or kainate in *ex vivo* slice preparations, within AD model mice is lacking. We examined gamma oscillations using field recordings in acute hippocampal slices from *5xFAD* and control mice, through the period of developing pathology, starting at 3 months of age, when there is minimal plaque presence in the hippocampus, through to 12+ months of age, when plaque burden is high. In addition, we examined PVIN participation in gamma rhythms using targeted cell-attached recordings of genetically-reported PVINs, in both wild type and mutant mice. In parallel, a developmental immunohistochemical characterisation probing the PVIN-associated expression of PV and perineuronal nets (PNNs) was compared between control and *5xFAD* mice. Remarkably, this comprehensive longitudinal evaluation failed to reveal any obvious correlations between PVIN deficits (electrical and molecular), circuit rhythmogenesis (gamma frequency and power), and Aβ deposits/plaque formation. By 6–12 months, *5xFAD* animals have extensive plaque formation throughout the hippocampus. However, a deficit in gamma oscillatory power was only evident in the oldest *5xFAD* animals (12+ months), and only when using kainate, and not carbachol, to induce the oscillations. We found no difference in PV firing or phase preference during kainate-induced oscillations in younger or older *5xFAD* mice compared to control, and a reduction of PV and PNNs only in the oldest *5xFAD* mice. The lack of a clear relationship between PVIN function, network rhythmicity, and plaque formation in our study highlights an unexpected resilience in PVIN function in the face of extensive plaque pathology associated with this model, calling into question the presumptive link between PVIN pathology and Alzheimer’s progression.

## Introduction

Alzheimer’s disease (AD) is a progressive, degenerative, neurological condition which has devastating effects on memory, personality and ultimately other cognitive and physical functions. The disease is most notably characterised by the presence of extracellular amyloid beta (Aβ) plaques and intracellular neurofibrillary tangles (NFT) containing hyperphosphorylated tau, and in later stages, extensive cell death leading to brain atrophy (Ogomori et al., 1989; Braak and Braak, 1991; Serrano-Pozo et al., 2011; Selkoe and Hardy, 2016; Lane et al., 2018).

In recent years there has been an interest in examining how different types of rhythmic brain network activities are affected in AD, especially in relation to cognitive decline seen in the disease. Gamma frequency oscillations (30–90 Hz) are of particular interest given that they have been recorded during cognitively demanding tasks in humans and other animals (Lisman and Idiart, 1995; Fries et al., 2001; Bauer et al., 2007; Colgin and Moser, 2010); and are thought to serve as a metric for higher-order cortical functions, and as a possible mechanism for cortical “binding” of the activity of distinct neuronal populations in regards to a common object of attention (Gray and Singer, 1989; Whittington et al., 1995, 2011; Singer, 1999; Draguhn and Buzsáki, 2004).

There have been a small number of studies which have looked at gamma oscillatory deficits in human patients with AD (Spydell and Sheer, 1983; Ribary et al., 1991; Başar et al., 2016); however, the majority of work has been done using mouse models of the disease. Gamma power has been reported to be reduced in a number of mouse models of AD *in vivo*: *PVJ20* mice and 8-month-old *J20* mice displayed reduced CA1 gamma power, recorded in awake behaving mice (Rubio et al., 2012; Etter et al., 2019); similarly, sharp-wave ripple (SWR)-associated decreases in gamma power were recorded in the CA1 of 3-month-old *5xFAD* mice *in vivo* (Iaccarino et al., 2016), and the dentate of aged *ApoE4* mice (Gillespie et al., 2016). *Ex vivo* slice experiments have also shown evidence of network dysfunction in AD with both kainate- and carbachol-induced gamma frequency power reductions seen in brain slices from transgenic mice, and in control brain slices exposed to different forms of Aβ (Driver et al., 2007; Kurudenkandy et al., 2014; Xiao et al., 2017; Arroyo-García et al., 2021).

In the CA3 region of the hippocampus, it is well established that the generation and coordination of gamma frequency oscillations is largely due to the activity of fast-spiking, PV-expressing interneurons (PVINs; Cobb et al., 1995; Whittington et al., 1995, 2011; McBain, 2001; Bartos et al., 2002; Gulyás et al., 2010). Impaired PVIN activity can result in disrupted brain network activity and hypersynchrony in various disorders including AD (Palop and Mucke, 2016). *In vitro* hippocampal slices from 3-month-old *5xFAD* mice displayed increased SWRs and reduced firing frequency of PV-expressing basket cells within CA1 during these network events (Caccavano et al., 2020). PVINs recorded in the parietal cortex of *hAPP J20* AD mice expressed lower levels of the voltage-gated Na channel, Na_V_1.1, and displayed smaller amplitude action potentials and impaired inhibitory synaptic transmission onto layer 2/3 parietal pyramidal cells. This loss in PVIN function also affected network dynamics, yielding reduced gamma frequency activity and coincident network hyperexcitability *in vivo*, which was restored by rescuing levels of Na_V_1.1 within PVINs (Verret et al., 2012). Similarly, transplants of Na_V_1.1-overexpressing PVINs enhanced AP kinetics and firing frequency, reduced hyperexcitability, and restored behaviour-modulated gamma oscillations in *hAPP J20* mice (Martinez-Losa et al., 2018). Furthermore, it is widely accepted that gamma frequency stimulation of PVINs is sufficient to induce gamma oscillations, whilst their targeted inhibition suppresses it, showcasing their important role in this form of network activity (Cardin et al., 2009; Sohal et al., 2009). This has led to a number of studies using optogenetic or auditory gamma frequency stimulation of PVINs to improve network and behavioural deficits and reduce plaque burden in a number of AD mouse models, highlighting the importance of studying PVINs with regard to network dysfunction in AD (Iaccarino et al., 2016; Lee et al., 2018; Etter et al., 2019; Martorell et al., 2019; Zhang et al., 2020).

Despite these findings, there is still a lack of knowledge concerning the onset or development of gamma deficits in the disease in relation to plaque burden, and limited comparison of gamma oscillatory activity induced by different mechanisms. Here we provide an in-depth, longitudinal assessment of gamma frequency network disruption, in a well characterised *ex vivo* brain slice model, in relation to PVIN deficits and plaque deposition. All of the experiments described in this manuscript were done using the *5xFAD Tg-6799* (*5xFAD*) model (Oakley et al., 2006). Although no model completely recapitulates the human phenotype of the disease, this multi-knock-in model exhibits accelerated plaque pathology, cognitive memory deficits, and synapse/cellular loss (Oakley et al., 2006; Jawhar et al., 2012; Schneider et al., 2014; Martorell et al., 2019). We recorded kainate (KA)- and carbachol (Cch)-induced gamma oscillations within mid-ventral hippocampal slices, as well as PVIN spiking activity during KA, from young and old, *5xFAD* and control mice. Brain slices were then processed for Aβ, PV and perineuronal net (PNN) quantification using immunohistochemistry. Our findings do not support a clear relationship between hippocampal plaque presence and impaired gamma oscillatory network activity as a result of PVIN dysfunction. Plaque formation developed as previously described for this model (Oakley et al., 2006), with minimal hippocampal plaque presence at 3–4 months progressing to a much higher plaque burden by 6–12 months throughout the hippocampus. However, despite extensive tissue pathology only KA- and not Cch-induced gamma frequency oscillations in the oldest *5xFAD* mice were found to be significantly impaired. PVIN firing during KA was not different between *5xFAD* and control mice, with the only differences observed between older and younger animals. Thus, the link between plaque pathology and network dysfunction is more complex than previously thought and this model demonstrates a surprising resilience against plaque pathology with regard to PVIN function and network rhythmogenesis.

## Materials and Methods

### Animals

All experiments were conducted in accordance with animal protocols approved by the National Institutes of Health.

For all electrophysiology and immunohistochemistry experiments, both male and female transgenic *5xFAD* mice were used [Jax MMRRC stock #34840, B6SJL-Tg (APP^SwFlLon^, PSEN1^*M146L*L286V^) 6799Vas]. Three to four month-old animals were used for the “young” timepoint. These were chosen due to the minimal hippocampal plaque development present at this stage (Oakley et al., 2006). For the “old” timepoints, 12–19-month-old animals were used (except with cell-attached, patch-clamp experiments where 10–13-month-old animals were used). At this stage, plaque deposition was extensive throughout the cortex and hippocampus. All animals were group-housed in individually ventilated cages kept at room temperature with a 12 h/12 h light/dark cycle and provided with food and water *ad libitum*.

All control experiments were done using littermate controls or PV-tdTomato mice for patching experiments. To label PV cells for targeted patch clamp experiments, *5xFAD* mice were crossed with PV-tdTomato mice maintained on a C57BL/6 background [Stock #027395, C57BL/6-Tg (Pvalb-tdTomato) 15 Gfng/J]. Mouse genotype was confirmed pre and post experiment by tail biopsy using Transnetyx genotyping services.

### Antibodies

Mouse anti-PV (1:5,000; Sigma-Aldrich, MO, USA Cat# P3088, RRID:AB_477329), mouse anti-beta amyloid (1:1,000; BioLegend, CA, USA Cat# 803004, RRID:AB_2715854) were used as indicated below. For PNN labelling biotinylated WFA (1:500, Vector Laboratories, CA, USA Cat#B-1355-2) was used as a primary and, Streptavidin Alexa 488 or 555 was used as a secondary (1:1,000; Thermo Fisher Scientific, MA, USA). Similarly, for the PV and beta amyloid, secondary antibodies were conjugated with Alexa Fluor dyes 488 or 555 (1:1,000; Thermo Fisher Scientific, MA, USA).

### Immunohistochemistry (IHC) on Drop Fixed Tissue

All immunohistochemical staining was performed on hippocampal slices used for the electrophysiology experiments. When the experimental recordings finished, slices (350 μm) were taken from the interface chamber and immediately drop fixed in 4% PFA for 4–24 h before being transferred to a 30% sucrose solution for >24 h. The slices were then re-sectioned using a freezing microtome to 70 μm and stored in a cryoprotective solution (25% glycerol, 30% ethylene glycol in PBS) at −20°C until required.

For immunostaining, sections were rinsed in PBS at room temperature then blocked for 2 h in 10% normal goat serum with 0.5% Triton X-100 then incubated in the primary antibody at 4°C for 24–48 h. Sections were then rinsed with PBS and incubated in secondary antibodies (1:1,000) for 2 h at room temperature. All antibodies were diluted in a carrier solution consisting of PBS with 1% BSA, 1% normal goat serum, and 0.5% Triton X-100. Sections were then rinsed, mounted on Superfrost glass slides, and cover slipped using prolong gold antifade mounting medium (Thermo Fisher Scientific; Cat# P-36931) and 1.5 mm cover glasses.

### Image Acquisition and Analysis

Confocal images were taken using a Zeiss 780 confocal microscope. Amyloid plaque density analysis was done using Imaris software (Oxford Instruments, RRID:SCR_007370). Plaque density was measured using the surfaces tool in Imaris on stitched, Z-stack images which were segmented into regions containing either the CA1 subfield or the dentate/CA3 with reference to the Paxinos and Franklin mouse brain atlas (Franklin and Paxinos, 2008). Plaque density was quantified as the volume of plaques per volume of analysed region and presented as a percentage density within CA1 or dentate/CA3. Quantitative analysis of PNN and PV+ cell density in the hippocampus was performed using ImageJ software (NIH, Bethesda, MD, USA, RRID:SCR_003070). The number of PV cells with/without PNNs, and PNNs without PV cells were counted and again normalised to the volume of the inspected region.

### Slice Preparation and Electrophysiology

Mice were anaesthetised with isoflurane until pedal withdrawal reflex was lost and then immediately decapitated. The brains were dissected in ice-cold sucrose artificial cerebrospinal fluid (SaCSF) containing the following (in mM): 90 sucrose, 80 NaCl, 3.5 KCl, 24 NaHCO_3_, 1.25 NaH_2_PO_4_, 4.5 MgCl_2_, 0.5 CaCl_2_, and 10 glucose, saturated with 95% O_2_ and 5% CO_2_. Horizontal brain slices (350 μm) were cut to obtain transverse sections of the mid-ventral hippocampus using a VT-1200S vibratome (Leica Microsystems, Wetzlar, Germany) and incubated in an interface-style chamber in normal aCSF solution containing (in mM): 130 NaCl, 3.5 KCl, 24 NaHCO3, 1.25 NaH_2_PO_4_, 2 MgCl_2_, 2 CaCl_2_ at 32–34°C for 30 min and then maintained at room temperature until use. This area was chosen for our experiments as it better preserves the structural hippocampal connectivity after slicing, thus enabling the generation of more robust gamma network activity than sections from the dorsal hippocampus (Steullet et al., 2010; Whitebirch, 2020). Slices were incubated for at least 1 h before conducting electrophysiological recordings.

For gamma oscillation experiments, all slices were transferred to a modified interface-style recording chamber (BSC-1, Scientific Systems design Inc., ON, Canada) and perfused with aCSF at 1.5–2 ml/min, 32–34°C. The field potentials were recorded using borosilicate glass micropipettes (World Precision Instruments, FL, USA) pulled to a resistance of 3–5 MΩ using a vertical pipette puller (Narishige, PP-830) and filled with aCSF and placed in CA3 *stratum pyramidale* and CA1 *stratum radiatum*. Signals were acquired at 10 kHz using a MultiClamp 700 A amplifier, filtered at 3 kHz (Molecular Devices, CA, USA), digitised using a Digidata1322A (Molecular Devices, CA, USA), and captured on a computer running pClamp9.2 (Molecular Devices, CA, USA). After a baseline of 5 min, gamma oscillations were induced by 10 μM carbachol (Sigma, MO, USA) or 400 nM kainate (Sigma, MO, USA cat# K0250).

For cell-attached recordings of PV cells, slices were transferred to a submerged recording chamber (Warner Instruments, CT, USA) with an upright microscope (Zeiss Axioskop) placed between two custom-made harps to allow perfusion above and below the slice with aCSF at 2–3 ml/min at 32°C. Similar to the interface recording set-up, the field potential was recorded from CA3 *stratum pyramidale* with a glass micropipette (3–5 MΩ). Individual PV cells were visualised using a 40× objective using fluorescence and IR-DIC camera microscopy. PV+ cells were located within the CA3 region and cell-attached recordings were made using 3–5 MΩ resistance microelectrodes filled with aCSF. Recordings were made using a Multiclamp 700 A amplifier (Molecular Devices, MO, USA), and signals were digitised at 10 kHz (Digidata 1322A, filtered at 3 kHz) for collection on a PC computer equipped with pClamp 9.2 or 10.4 software (Molecular Devices, CA, USA RRID:SCR_011323) Gamma oscillations were induced in submerged recording configuration using application of 100 nM KA (Sigma, MO, USA cat# K0250).

### Data Analysis

Pre-processing of electrophysiology data was done in Clampfit 10.5/11.2 (Molecular Devices, RRID:SCR_011323).

For gamma oscillations recorded from the interface chamber, all analysis was performed during the first 5 min during washout (where oscillations yielded the greatest power) after at least 30 min of drug application to allow time for the oscillation to build up. The signals were bandpass filtered between 5 and 120 Hz and then 10 s epochs were used for calculating the power spectrum, autocorrelation, and cross-correlation between CA3 and CA1. Slices were only included for analysis if the frequency at peak power (calculated from the power spectrum analysis) was >19 Hz and <80 Hz. Similarly, recordings were only included for analysis if the peak gamma power was >1 × 10^0^ μV^2^/Hz for CA3 and >1 × 10^-1^μV^2^/Hz for CA1. Recordings with significant mains noise contamination (~60 Hz) were also discarded. Peak gamma power, frequency at peak power, and bandwidth power (between 20 and 80 Hz) were extracted from power spectrum output. Although the frequency range for gamma oscillations is normally reported between 30 and 90 Hz, the optimal recording temperature for our experimental set-up was 5–7°C lower than body temperature and due to the linear relationship between recording temperature and oscillation frequency, we shifted the limits for our bandwidth power calculations to 20–80 Hz to account for this (Dickinson et al., 2003; Schneider et al., 2015). Cross-correlation peak and cross-correlation delay between CA3 and CA1 were obtained from the Clampfit cross-correlation output. The rhythmicity of the gamma oscillations was determined by calculating the coefficient of rhythmicity from the autocorrelograms (Cangiano and Grillner, 2003; Balleza-Tapia et al., 2018). Briefly, the value was defined as Cr = (α−β)/(α+β) where α and β are the height of the second peak and the first trough in the normalised autocorrelogram, respectively. The values ranged from 0 to 1 with all values for our data found to be ≥0.01 and therefore considered rhythmic. The higher the Cr values the better the quality of the oscillations.

Pre-processing of PVIN spike data was done using the threshold detection tool in Clampfit. Three minute epochs were selected for analysis at baseline, during kainate, and in drug washout. Spike analysis from the threshold detection was exported along with the respective section of the abf file trace and used for phase timing analysis in MATLAB using custom-written code (Caccavano et al., 2020). For slices that developed ictal-like activity, abf files were analysed using MATLAB to examine PVIN spike half-width and frequency during interictal bursting.

### Statistical Analysis

All data were assessed for normality and then tested with appropriate parametric or non-parametric tests using GraphPad Prism (GraphPad Prism 9.1.0, RRID:SCR_002798) or MATLAB (MATLAB R_2020, RRID:SCR_001622). Where data displayed lognormal distribution, they were log-transformed prior to running any statistical tests. Plots are represented as box and whisker plots with the median, 25th–75th percentile, and range of data shown. Several results are presented in a two-way design, with age and genotype as the main factors. These were analysed by a two-way ANOVA including the interaction. For consistency between endpoints, the *post-hoc* tests were kept consistent and based on *a priori* hypotheses (specifically: young control vs. young *5xFAD*, old control vs. old *5xFAD*, old control vs. young control, and old *5xFAD* vs. young *5xFAD*). For transparency, all results from the two-way ANOVA and *post-hoc* tests are presented in the Supplementary Stats File. PVIN firing data were analysed by fitting a mixed model, as implemented in GraphPad Prism 9.0, instead of a repeated measures ANOVA which cannot handle missing values. This mixed model used a compound symmetry covariance matrix fit using Restricted Maximum Likelihood (REML). Circular data from the phase-locking analysis were analysed in MATLAB. First-order cellular data were tested for non-uniformity using Raleigh’s test to determine the significance of phase-locking with the Circular Statistics Toolbox for MATLAB (RRID:SCR_016651; Berens, 2009). Second-order circular data of the population of cells (including those not significantly phase-locked) were analysed with a custom MATLAB implementation of Hotelling’s and Batschelet’s methods to compute the weighted mean vector and significance of one and two-sample means (Sections 26.9, 27.9, 27.11 of Zar, 2010). All data points on box plots indicate the individual or average values for a single slice or single cell. P values displayed in plots in bold or with ^*^ indicate significance. *p < 0.05, **p < 0.01, ***p < 0.001 and ****p < 0.0001. Figures were made in MATLAB, GraphPad Prism, and Inkscape (Inkskape 1.0, RRID:SCR_014479).

## Results

### Plaque Presence in *5xFAD* Model

**Figure 1:**
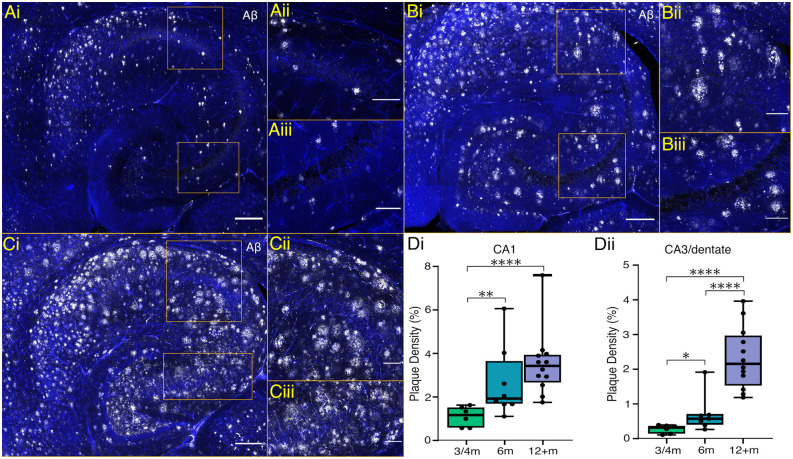
Hippocampal plaque development in *5xFAD* mice. **(Ai,Bi,Ci)** Representative maximum intensity projection images of ventral hippocampal slices processed with an anti-amyloid beta antibody (Aβ; white) and DAPI stain (blue) from 4, 6 and 12-month-old mice, respectively (scale bar = 250 μm). **(Aii,Bii,Cii)** Enlarged sections of CA1 plaque presence and **(Aiii,Biii,Ciii)** of CA3 (scale bar = 100 μm). **(D)** The plaque densities as a percentage of measured volumes are shown from CA1 **(Di)** and CA3/dentate **(Dii)** across timepoints. Box plots show the median as a black line, boxed region as the interquartile range, and whiskers as the range with individual points representing data from one brain slice. 3–4 m: *n* = 6 slices from four mice (two female); 6 m: *n* = 8 slices from four mice (two females); 12+ m: *n* = 12 slices from six mice (three females). *p < 0.05, **p < 0.01, ****p < 0.0001 (one-way ANOVA with Šidák correction).

*5xFAD* mice display accelerated Aβ plaque development with extracellular deposits, appearing in deep cortical layers and subiculum by 2 months, and spreading to other brain regions, including the hippocampus, by 4 months (Oakley et al., 2006). We confirmed the presence of extracellular Aβ deposits at 3 and 4 months within the cortex and subiculum as well as some minor plaque deposition in the hippocampus (Figure 1). Whilst labelling of Aβ deposits was seen throughout the subiculum at 3–4 months, within the hippocampus labelling was sparser, with no deposits in the pyramidal and granule cell layers and most plaque presence occurring in the surrounding layers e.g., *stratum oriens* and *stratum radiatum* of CA1 and CA3, and the hilar region of the dentate (Figure 1A). At 6 months, plaque density significantly increased, particularly in the CA1 subfield (Figures 1B,D). In tissue from animals 12 months or older, Aβ plaque deposition occurred throughout the hippocampal formation with plaques encroaching on the previously spared granule and pyramidal cell layers (Figures 1C,D).

### Carbachol (Cch)-Induced Gamma Frequency Oscillations

**Figure 2:**
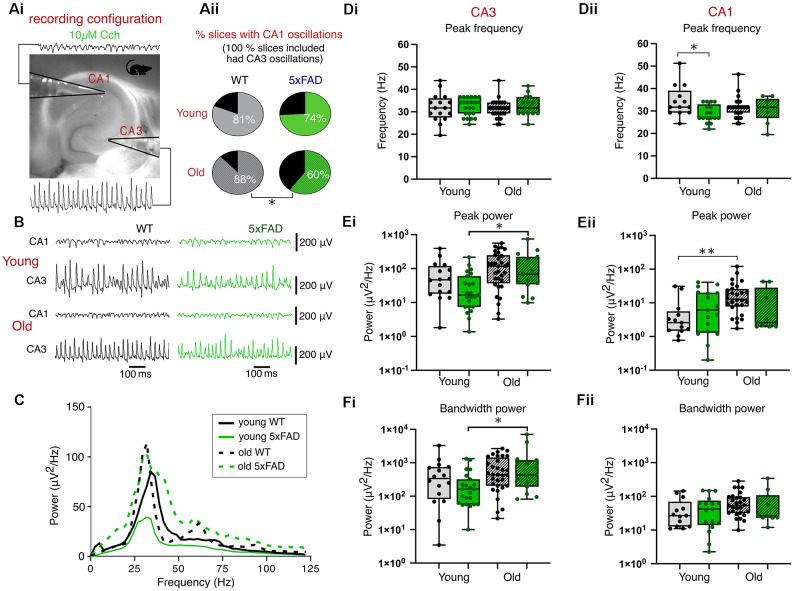
Carbachol-induced gamma oscillations. **(Ai)** Experimental configuration used for recording gamma oscillations in an interface recording chamber. **(Aii)** Pie charts indicating the percentage of slices with coincident CA1 and CA3 gamma oscillations (all slices included in analysis exhibited CA3 oscillations). **(B)** Representative traces of carbachol-induced gamma oscillations. **(C)** Average CA3 power spectrums of young and old, control and *5xFAD* slices during carbachol-induced oscillations. **(D,E,F)** Group data boxplots of the peak frequency, peak gamma power and bandwidth power respectively from young and old, control and *5xFAD* animals in CA3 **(Di,Ei,Fi)** and CA1 **(Dii,Eii,Fii)**. Box plots show the median as the black line, the boxed region as the interquartile range, and the whiskers as the range with each data point representing a single slice. Young WT: *n* = 16/13 (CA3/CA1), eight mice (three females); Young *5xFAD*: *n* = 23/17 (CA3/CA1), eight mice (four females); Old WT: *n* = 32/28 (CA3/CA1), six mice (one female); Old *5xFAD*: *n* = 15/9 (CA3/CA1), five mice (two females). *p < 0.05, **p < 0.01 (**Aii**: χ^2^ test; **D**,**E**,**F**: two-way ANOVA with Šidák correction for multiple comparisons).

To study the effect of developing plaque presence on network activity within the hippocampus, we compared gamma frequency oscillations in *ex vivo* brain slices from *5xFAD* and control mice (Figure 2A). This form of rhythmic activity is commonly induced by the perfusion of the cholinergic agonist, carbachol (Cch), which yields persistent oscillatory activity around 40 Hz within CA3, and propagates to CA1 (Fisahn et al., 1998). Figure 2 shows that the application of 10 μM Cch successfully induced CA3 and CA1 gamma frequency oscillations in young and old control and *5xFAD* brain slices (Figures 2B,C). The peak CA3-recorded gamma frequency did not differ between control slices and *5xFAD* brain slices from young or old mice (Figure 2Di). Gamma power (Figure 2Ei) and bandwidth power (Figure 2F) were also not different between control and *5xFAD* brain slices, however, the two-way ANOVA showed significant age effects with *post-hoc* tests indicating significant increases in CA3 power and bandwidth power in older *5xFAD* brain slices (Figures 2Ei,Fi).

The presence of cholinergically-induced gamma oscillations in the CA1 is dependent upon intact circuitry and communication from CA3 *via* the Schaffer collaterals and so we also recorded from CA1 *stratum radiatum*, where it is possible to detect gamma oscillations, albeit at lower power than those of CA3 (Fisahn et al., 1998; Zemankovics et al., 2013). All slices used in our analysis exhibited CA3 gamma oscillations, however, we found a difference between WT and *5xFAD* mice in the proportion of slices which exhibited coincident CA1 gamma oscillations. In slices from younger mice, 82% of control slices compared with 74% of *5xFAD* slices had both CA3 and CA1 gamma frequency activity (*p* = 0.59, χ^2^ test; Figure 2Aii). In slices from the older cohort of mice, this difference was more pronounced with 88% of control slices exhibiting gamma oscillations in both CA3 and CA1 as opposed to only 60% of *5xFAD* slices (*p* = 0.03, χ^2^test; Figure 2Aii). Despite differences in the proportion of slices exhibiting coincident CA1 gamma frequency oscillatory activity between *5xFAD* and control tissue, in slices that did show CA1 gamma oscillations, the results of the two-way ANOVA showed no significant effect of genotype on CA1 power (Figure 2Eii) or bandwidth power (Figure 2Fii). There was a significant effect of genotype on CA1 frequency, which after *post-hoc* tests showed a significant decrease in peak gamma frequency in young *5xFAD* slices (Figure 2Dii). The two-way ANOVA also showed a significant age effect for CA1 gamma power which, after post-hoc tests, revealed a significant increase in CA1 power in older compared to young WT slices (Figure 2Eii).

### Rhythmicity and Synchronicity of Cch-Induced Gamma Oscillations

**Figure 3:**
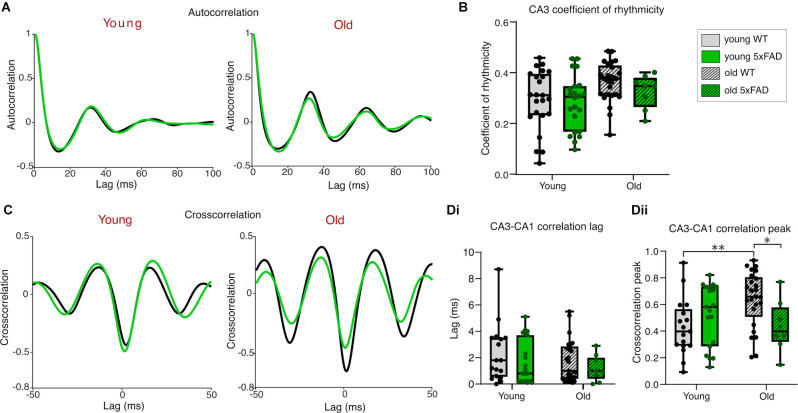
Rhythmicityof carbachol-induced gamma oscillations. **(A)** Mean autocorrelograms from CA3 recorded gamma oscillations. **(B)**Boxplots of the calculated coefficient of rhythmicity of the oscillations (see Section “Materials and Methods” for details of calculation). **(C)** Mean cross correlograms of CA3-CA1. **(D)** Group data cross correlation lags **(Di)** and peaks **(Dii)** from CA3 to CA1. Young WT: *n* = 23/17 **(A,B/C,D)**, eight mice (three females); Young *5xFAD*: *n* = 23/19 **(A,B/C,D)**, eight mice (four females); Old WT: *n* = 25/25 **(A,B/C,D)**, six mice (one female); Old *5xFAD*: *n* = 8/9 **(A,B/C,D)**, five mice (two females). *p < 0.05, **p < 0.01 (two-way ANOVA with Šidák correction for multiple comparisons).

We calculated the coefficient of rhythmicity for CA3 gamma oscillations in young and old *5xFAD* and control tissue to investigate the quality of the generated gamma oscillations (see Section “Materials and Methods”). The average CA3 autocorrelograms for each condition are shown in Figure 3A and despite a significant age-effect indicated by the two-way ANOVA, *post-hoc* tests revealed no differences in rhythmicity between young and old, control or *5xFAD* oscillating brain slices (Figure 3B).

The precise coordination of activity between different subregions of the hippocampus is crucial for proper information transfer and memory consolidation (Kanak et al., 2013). For this reason, we examined the cross-correlation between CA3 and CA1 in the young and old *5xFAD* and control mouse tissue (Figures 3C,D). The phase lags measured from the cross-correlation peaks were no different between young and old, or control and *5xFAD* slices (Figure 3Di), suggesting that the Schaffer collateral pathway is not selectively impaired in *5xFAD* slices from young or old mice. A two-way ANOVA comparing the peak cross-correlation values between CA3 and CA1 showed a significant interaction effect with *post-hoc* testing revealing higher cross-correlation in older compared to younger WT slices. This age-related increase in CA3-CA1 coordination was not seen in the *5xFAD* brain slices, where *post-hoc* testing showed significantly lower peak cross correlation than in age-matched WT brain slices (Figure 3Dii). This decrease in the peak cross-correlation in older *5xFAD* slices, without any genotype difference in the propagation delay between CA3 and CA1, may reflect impaired post-synaptic responsiveness or synchronicity within CA1 to CA3 rhythmic input despite intact Schaffer collateral afferents in the *5xFAD* tissue.

### Kainate (KA)-Induced Gamma Frequency Oscillations

Despite extensive plaque burden at 12 months and older, *5xFAD* slices exhibited remarkable resilience in their ability to generate robust Cch-induced gamma oscillations, with only modest impairments in rhythmicity and CA1 participation. There are many ways of inducing gamma frequency oscillations in brain slices, all of which function to increase excitability within pyramidal cells and/or interneurons (Fisahn, 2005). However, different methods work through specific targets and exhibit distinct properties. For example, while cholinergically-induced oscillations work through targeting M1 muscarinic acetylcholine receptors and require AMPA and GABA_A_ receptors (Fisahn et al., 1998, 2002), glutamatergically-induced gamma frequency oscillations, using kainate (KA), require KA and GABA_A_ receptors but not AMPARs (Fisahn et al., 2004; Fisahn, 2005). This demonstrates that whilst different pharmacological methods can produce similar network activity, they do so through different mechanisms and possibly involving different cell ensembles. Therefore, we next investigated if the network resiliency of *5xFAD* mice extends to KA-induced gamma oscillations.

**Figure 4:**
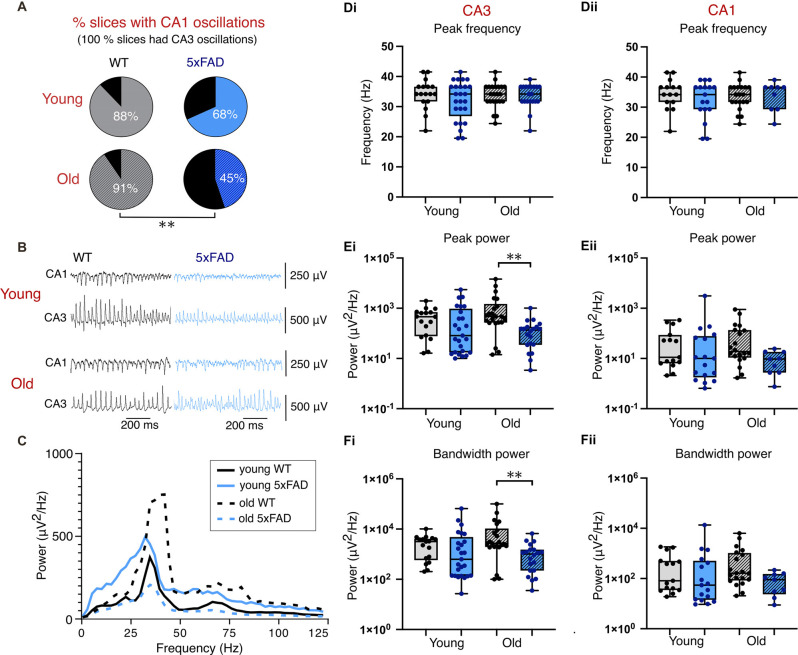
Kainate-induced gamma oscillations. **(A)** Pie charts indicating the percentage of slices with coincident CA1 and CA3 oscillations (all slices included in analysis exhibited CA3 gamma oscillations). **(B)** Representative traces of kainate-induced gamma oscillations. **(C)** Average CA3 power spectrums of young and old, control and *5xFAD* slices during kainate-induced oscillations. **(D,E,F)** Group data boxplots of the peak frequency, peak gamma power and bandwidth power, respectively, from young and old control and *5xFAD* animals in CA3 **(Di,Ei,Fi)** and CA1 **(Dii,Eii,Fii)**. Each data point represents a single slice. Young WT: *n* = 17/15 (CA3/CA1), eight mice (three female); Young *5xFAD*: *n* = 25/17 (CA3/CA1), eight mice (four females); old WT: *n* = 22/20 (CA3/CA1), eight animals (three females); old *5xFAD*: *n* = 20/9 (CA3/CA1), seven mice (three females). **p < 0.01 (**A**: χ^2^ test; **D**,**E**,**F**: two-way ANOVA with Šidák correction for multiple comparisons).

Notably, the application of 400 nM KA-induced higher power gamma oscillations than Cch in all conditions (Figures 4B,C). The frequency of CA3 gamma oscillations in KA was no different between control and *5xFAD*, or between age groups (Figure 4Di). Similarly, the CA3 power and bandwidth power did not differ between any groups (Figures 4Ei,Fi). However, in contrast to oscillations induced by Cch, KA-induced oscillations showed significant genotype effects for CA3 power and CA3 bandwidth power. After *post-hoc* testing, this revealed significant reductions in CA3 power and bandwidth power in older *5xFAD* brain slices compared to controls (Figures 4Ei,Fi).

Once again, 100% slices used in the analysis exhibited CA3 gamma oscillations and we found that, as with slices exposed to Cch, young and old *5xFAD* brain slices exposed to KA displayed lower CA1 participation in gamma frequency oscillations (Figure 4A). In slices from younger mice, 88% of control slices compared with 68% of *5xFAD* slices had coincident CA3 and CA1 gamma oscillatory activity (*p* = 0.085, χ^2^ test; Figure 4A). In slices from the older cohort of mice, the difference in CA1 participation was even more substantial with 91% of control slices exhibiting gamma oscillations in both CA3 and CA1 as opposed to only 45% of *5xFAD* slices (*p* = 0.001, χ^2^ test; Figure 4A). Despite lower CA1 participation in *5xFAD* brain slices, those which did exhibit CA1 gamma oscillations exhibited no difference in frequency, power, or bandwidth power after *post-hoc* testing, despite a significant effect of genotype in CA1 power (Figures 4Dii,Fii).

### Rhythmicity and Synchronicity of KA-Induced Gamma Oscillations

**Figure 5:**
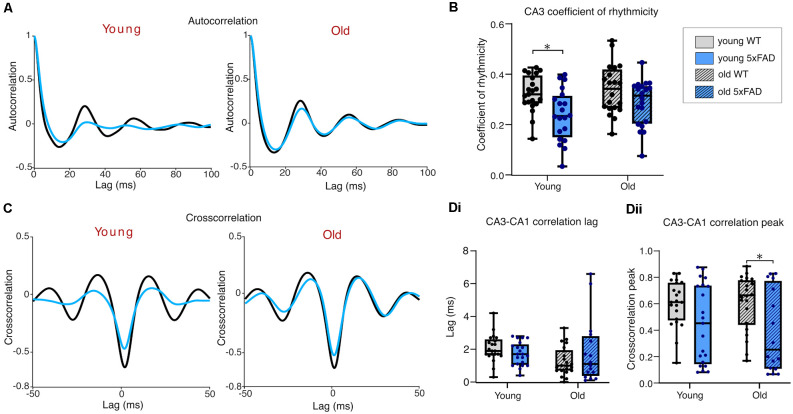
Rhythmicity of kainate-induced gamma oscillations. **(A)** Mean autocorrelograms from CA3 recorded gamma oscillations. **(B)** Boxplots of the calculated coefficient of rhythmicity of the oscillations. **(C)** Mean crosscorrelograms of CA3-CA1. **(D)** Group data cross correlation lags **(Di)** and peaks **(Dii)** from CA3 to CA1. Young WT: *n* = 21/21 **(A,B/C,D)**, eight mice (three females); Young *5xFAD*: *n* = 20/21 **(A,B/C,D)**, eight mice (four females); old WT: *n* = 20/22 **(A,B/C,D)**, eight mice (three females); old *5xFAD*: *n* = 20/16 **(A,B/C,D)**, seven mice (three females). *p < 0.05 (two-way ANOVA with Šidák correction for multiple comparisons).

Figure 5A shows the average autocorrelograms for KA-induced gamma oscillations in control and *5xFAD* slices, for young and old mice. The coefficient of rhythmicity (Cr) was similar across age groups; however, a significant genotype effect showed the Cr in young *5xFAD* slices to be significantly lower compared to controls after *post-hoc* testing (Figure 5B).

Cross-correlation phase lag between CA3 and CA1 was not different between *5xFAD* and control in either young or old brain slices (Figures 5C,Di). However, there was a significant genotype effect on peak cross-correlation, with old *5xFAD* slices exhibiting significantly reduced CA3-CA1cross-correlation compared to controls, after *post-hoc* testing (Figure 5Dii). In summary, our KA-induced gamma findings reveal poorer quality CA3 gamma oscillations and less CA1 participation, and at later stages, impaired synchrony between CA3 and CA1 and compromised gamma power in *5xFAD* mice.

### PVIN Firing Properties During KA-Induced Gamma Oscillations

As outlined above, the generation and coordination of gamma frequency oscillations are critically dependent upon the activity of fast-spiking PV-expressing interneurons (PVINs). Thus, based on the deficits observed in KA-induced oscillations in *5xFAD* slices, we chose to investigate PVIN function during KA-induced gamma oscillations in slices from young and old, *5xFAD*, and control mice.

**Figure 6:**
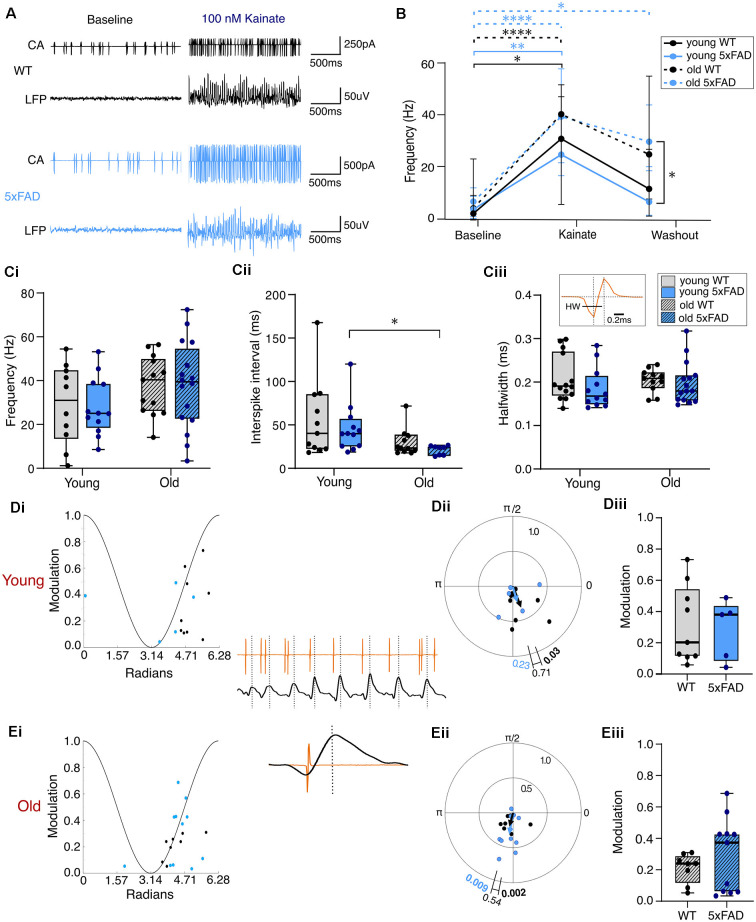
PVIN firing properties during kainate-induced gamma oscillations. **(A)** Representative traces of simultaneously recorded PVIN firing (cell-attached, CA, recordings) and extracellular CA3 local field potential (LFP) in baseline and then during kainate application. **(B)** Progressive change in PVIN firing frequency during the wash in and wash out of kainate plotted as the median with error bars indicating the 95% confidence interval, solid line for young, and dashed line for old data. **(Ci,Cii,Ciii)** Spike frequency, inter-spike interval, and spike halfwidth of PVINs during kainate, respectively. The inset in **(Ciii)** shows a representative PVIN action potential with the halfwidth shown with scale bar for reference. **(D,E)** Phase-locking of the PVIN spikes in relation to the gamma oscillation with the peak of the cycle at 0 and trough at π in young **(D)** and old slices **(E)**. The angles of individual data points in **(Di)** and **(Ei)** represent the mean phase angle for a single PV cell. The vector length/modulation is a value between 0 and 1 indicating the degree of phase-locking with a value of 1 meaning the cell always fires at the same phase and a value of 0 meaning the cell fires randomly with no phase-locking. The lines on the polar plots **(Dii,Eii)** indicate the weighted average degree of phase locking for the population of cells (see Section “Materials and Methods” for details). The p values shown at the end of each line on the polar plot indicate the results from Hotelling’s test of one-sample weighted means and the p-value shown above the bracket indicates the result of Batschelet’s test between the group population means. The insets show a representative trace of PVIN phase-locked spiking (in orange) to the oscillating LFP (black) with an enlarged view of a single phase-locked PVIN spike shown below. **(Diii,Eiii)** Box plots of the population data of the strength of phase modulation (median = black line, boxed region = interquartile range, and whiskers = full range). **(B,C)** Young WT: *n* = 10 cells, four mice (two female); Young *5xFAD*: *n* = 12 cells, three mice (all males); Old WT: *n* = 13 cells, four mice (all males); Old *5xFAD*: = 16 cells, three mice (two females). **(D,E)** Young WT: *n* = 9 cells, three mice (one female); Young *5xFAD*: *n* = 5 cells, two mice (all males); Old WT: *n* = 8 cells, three mice (all male); Old *5xFAD*: *n* = 11 cells, three mice (two females). *p < 0.01, **p < 0.01, ****p < 0.0001 [for **(B)** we used Repeated-measures Mixed-model approach with Šidák correction for multiple comparisons; **(C,D)** used two-way ANOVA with Šidák correction for multiple comparisons].

In submerged slices (see Section “Materials and Methods” and Hájos et al., 2009), application of 100 nM KA initiated gamma oscillations and results from the mixed model analysis and *post-hoc* testing showed significantly increased PVIN firing frequency in all groups upon kainate application (Figures 6A,B). This analysis also showed no effect of genotype on firing frequency (Figure 6Ci). Similarly, the results from two-way ANOVAs showed no genotype effect for inter-spike interval (ISI) or PVIN spike half width during KA-induced gamma oscillations (Figure 6Cii,iii). Interestingly, despite no observed genotype-specific differences in the behaviour of PVIN firing during KA application, the mixed model analysis revealed a significant effect of age as well as condition on PVIN firing frequencies. There was a non-significant trend for older PVINs from both genotypes to fire at higher frequencies during KA application, and a failure to return to lower levels of firing during the washout period, which was significantly different between young and old *5xFAD* slices after *post-hoc* tests (Figures 6B,Ci). The inter-spike interval also showed a significant effect of age during kainate application, with a significant reduction in old compared to young *5xFAD* PVINs after *post-hoc* tests (Figure 6Cii).

### Phase-Locking of PVINs During Kainate-Induced Gamma Oscillations

As the KA-induced gamma deficits in *5xFAD* brain slices appear unrelated to differences in the overall firing rates of PVINs, we probed for differences in phase-locking of PVINs to the gamma rhythm. Impaired phase-locking of fast-spiking interneurons (FSINs) to gamma frequency oscillations has been reported in APP^NL-G-F^ AD mice prior to plaque formation as well as in FSINs after exposure to Aβ_42_ application (Andrade-Talavera et al., 2020; Chung et al., 2020; Arroyo-García et al., 2021). Furthermore, recordings at reciprocal PC-PVIN synapses show both reduced EPSCs onto PVINs and reduced IPSCs and increased paired pulse depression at PVIN-PC synapses after treatment with Aβ_1–42_ oligomers (Chung et al., 2020; Park et al., 2020).

Despite this evidence of Aβ-induced synaptic disruption at both PC-PVIN and PVIN-PC synapses, we did not find any noticeable deficit in PVIN-gamma phase-locking in brain slices from *5xFAD* mice. Of the subset of PVIN recordings with detectable LFP gamma oscillations (submerged recording configuration), almost all PVINs fired with an average phase corresponding to the rising phase of the gamma cycle (Figures 6D,E). A first order analysis of PVIN spiking activity revealed significant phase-locking for most cells (young control: eight of nine; young *5xFAD*: five of five; old control: seven of eight; old *5xFAD*: nine of 11; *p* < 0.05, Raleigh’s test). A second order analysis of the population found significant gamma phase-locking of PVINs from young control mice at 5.070 rad/290° (*p* = 0.0266, Hotelling’s test), but no significant phase-locking of PVINs from young *5xFAD* brain slices, which displayed an average phase of 4.958 rad/284° (*p* = 0.2349, Hotelling’s test). However, this is likely related to low sampling in the mutant rather than a real-genotype related loss of the ability of PVINs to significantly phase-lock to gamma. There was no genotype difference between the mean phase angles (*p* = 0.7123, Batschelet’s test; Figure 6Dii). Older PVINs from both control and *5xFAD* brain slices were significantly phase-locked to the gamma oscillation (Control = 4.450 rad/255°; *p* = 0.0085, Hotelling’s test. *5xFAD* = 4.539 rad/260°; *p* = 0.0221, Hotelling’s test). There was no genotype difference between the mean phase angles (*p* = 0.5402, Batschelet’s test; Figure 6Eii). There was also no significant difference between young or old *5xFAD* and control PVINs with regards to the strength of modulation (Figures 6Diii,Eiii). However, the modulation variance was significantly greater in PVINs from older *5xFAD* mice (*p* = 0.0242, *F*_(10, 7)_ = 6.19), which could potentially point to differential alterations in PVIN subpopulations. To further explore this, a Kolmogorov-Smirnov analysis of the strength of modulation was conducted but revealed no significant alterations in the underlying distributions in either young (*p* = 0.9461, *D* = 0.244) or old (*p* = 0.1271, *D* = 0.5455) mice.

### KA-Induced Hyperexcitability

**Figure 7:**
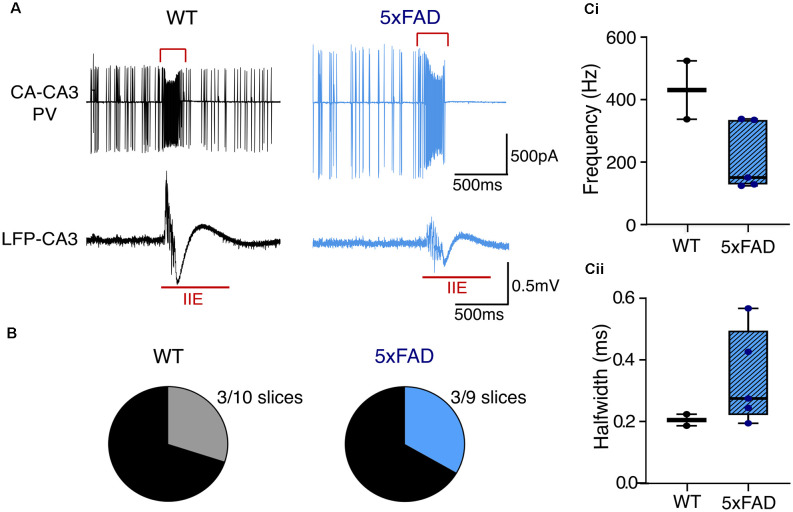
Ictal-like activity patterns in *5xFAD* and control slices during kainateapplication. **(A)** Representative traces from brain slice recordings in kainate that developed ictal-like activity. The top traces show the cell-attached PVIN recordings, and the bottom traces show the simultaneously recorded LFP during an inter-ictal event (shown in red)—the red square bracket in the PVIN recording indicates the section used for the analysis of spike properties during the IIE. **(B)** The total number of slices in submerged configuration from older WT and *5xFAD* mice that displayed interictal-like activity. WT: *n* = 10 slices, three mice (zero female); *5xFAD*: *n* = 9 slices, three mice (two females). **(C)** Boxplots of the PVIN firing frequency **(Ci)** and spike halfwidth **(Cii)** during the IIEs, from a subset of recordings which displayed both interictal activity and PVIN spiking. WT: *n* = 2 slices, one male mouse; *5xFAD: n* = 5 slices, three mice (one female). The median is shown by the black line and the boxed region indicates the interquartile range with the range shown by the whiskers [Data in **(C)** Mann-Whitney U test].

Network hyperexcitability is commonly comorbid with AD (Bakker et al., 2012; Palop and Mucke, 2016; Ambrad Giovannetti and Fuhrmann, 2019). Likewise, different AD models exhibit network hyperexcitability and increased predisposition to ictal-like activity (Busche et al., 2008; Sanchez et al., 2012; Verret et al., 2012; Siwek et al., 2015; Ciccone et al., 2019). We were therefore interested in evaluating any differences between *5xFAD* and control slices in their propensity for hyperexcitability. A number of slices from the older mice developed ictal-like activity during KA application in the submerged recording configuration (Figures 7A,B). There was no difference in the percentage of slices that developed ictal-like activity (3/10 of WT recordings, 30%; 3/9 of *5xFAD* recordings, 33.3%). There was also no significant difference in the PVIN firing frequency or spike half width during interictal events (IIEs), despite a trend towards lower frequency firing in the *5xFAD* PVINs. However, this could also be due to the low sampling size, as only a subset of the slice recordings exhibiting ictal-like activity had paired PVIN activity and could be used for analysis of IIE-spike properties.

### PV and Perineuronal Net (PNN) Presence in Young and Aged *5xFAD* and Control Mice

**Figure 8:**
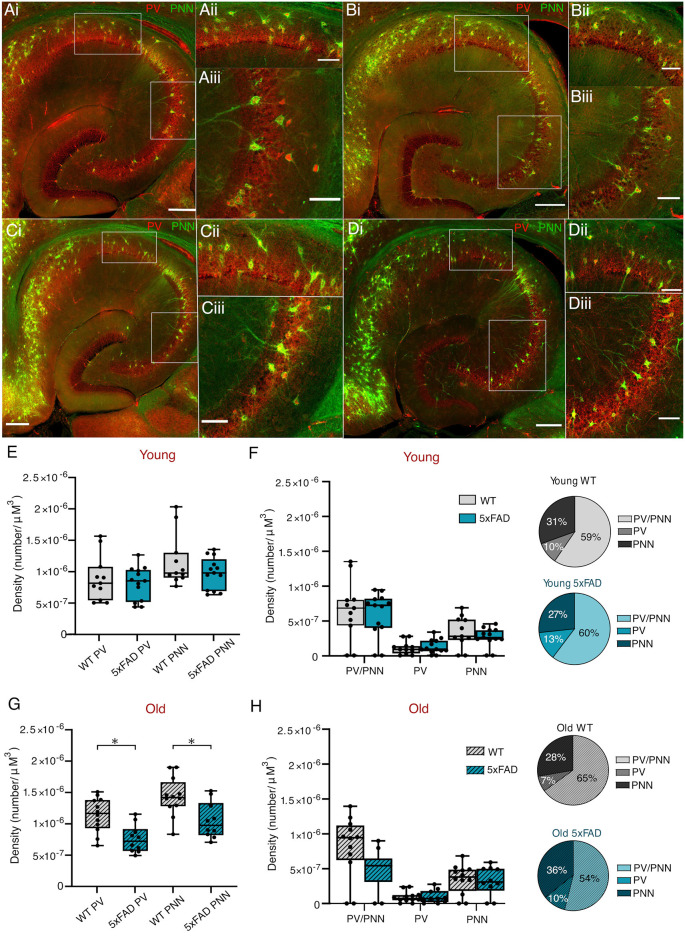
PVIN and PNNdensities in young and old *5xFAD* and control mice.**(Ai–Di)** Representative maximum intensityprojection images of ventral hippocampal slices processed withanti-PV and biotinylated WFA antibodies to label PV cells(red) and perineuronal nets (PNNs; green), respectively (scalebar = 250 μm). Young control **(A)**, Young *5xFAD***(B)**, Old control **(C)**, Old *5xFAD***(D)**. Enlarged images of the CA1**(Aii–Dii)** and CA3**(Aiii–Diii)** regions highlighted in the boxed region on the main image (Scale bar = 100 μm). **(E,G)** Total PV numbers and PNN numbers in young and old slices within CA1 and CA3. **(F,H)** Combined CA1/CA3 density of PV cells with PNN, PV cells without PNN, and PNN without PV cells in the young and old slices, respectively, with the proportions shown additionally as pie charts. Box plots show the median as the black line, the boxed region indicates the interquartile range, and the whiskers indicate the range. The individual data points represent slices. Young WT: *n* = 11 slices, four mice (two females); Young *5xFAD*: *n* = 13, three mice (two females); old WT: *n* = 12 slices, six mice (three females); old *5xFAD*: *n* = 10 slices, six mice (three females). *p <0.05 (two-way ANOVA with Šidák correction for multiple comparisons).

Accounts of whether interneuron numbers are affected in AD are controversial, with many contradicting reports (Morawski et al., 2010; Mahar et al., 2017; Cattaud et al., 2018; Ambrad Giovannetti and Fuhrmann, 2019; Hongo et al., 2020). Likewise, there is uncertainty as to whether or not there is any disease-related deficit in perineuronal nets (PNNs), the extracellular matrix structures which preferentially surround PVINs and promote their functioning (Morawski et al., 2010; Cattaud et al., 2018; Hayani et al., 2018; Crapser et al., 2020). We compared numbers of PVINs with and without PNNs, in control and *5xFAD* mice throughout disease progression, to determine if the mutants exhibited any differences that correlated with the oscillatory deficits observed (Figures 8A–D). The results of a two-way ANOVA showed a significant effect of age on PVIN numbers, and of genotype on PNN numbers. After *post-hoc* testing, this revealed a significant reduction in the total numbers of PVINs and PNNs from older *5xFAD* brain slices (Figure 8G), with no differences in younger WT and *5xFAD* mice (Figures 8E,F). This appears to be due to a non-significant reduction in older *5xFAD* PVINs and their associated PNNs (Figure 8H).

## Discussion

In this study, we investigated potential correlations in the hippocampal plaque development and network activity over time in the *5xFAD* model of AD focusing on two distinct time points (3–4 months and 12+ months), each with distinct hippocampal plaque burdens (Figure 1). This allowed for an in-depth approach to investigating network and PVIN function in relation to plaque pathology using widely published models of *in vitro* gamma oscillations. We found that gamma frequency oscillations are differentially affected in brain slices from *5xFAD* mice depending on the pharmacological approach used to initiate the oscillations. KA-induced oscillations showed significant power deficits in older *5xFAD* slices (Figure 4) and impaired rhythmicity in young (3–4 months) and old (12+ months) *5xFAD* slices (Figure 5). In contrast, Cch-induced oscillations showed no power or frequency deficits (Figure 2) and only minor rhythmicity deficits in the oldest *5xFAD* slices (Figure 3). We additionally looked at the function of PVINs during gamma frequency oscillations and found that their activity remained remarkably stable when compared between *5xFAD* and control mice (Figure 6). However, there was a trend towards increased PVIN excitability in older tissue, with older PVINs firing more frequently during KA application and washout compared to younger PVINs (Figure 6). Although PVIN function was conserved, numbers of PVINs and their associated PNNs (supportive extracellular matrix structures around PVINs) were significantly reduced in older *5xFAD* brain slices (Figure 8). Further work is required to ascertain whether the PVIN loss is Aβ plaque-mediated or caused by other disease mechanisms such as excitotoxicity. Regardless, impaired network activity was only seen during KA-induced oscillations, despite significant reductions in PVINs and extensive plaque deposition revealing a remarkable degree of network level resilience for patterned activity in the *5xFAD* mouse model.

### Gamma Frequency Disruption in Alzheimer’s Disease

Gamma oscillation deficits have often been implicated in the cognitive and memory decline seen in AD patients (Spydell and Sheer, 1983; Ribary et al., 1991; Hermann et al., 2009; Başar et al., 2016). AD mouse models have provided some potential insights into the network mechanisms underlying gamma frequency impairments observed in human EEG recordings. Indeed, a number of studies in various AD mouse models have also reported gamma deficits in *ex vivo* slice preparations, and *in vivo* recordings (Driver et al., 2007; Iaccarino et al., 2016; Etter et al., 2019; Zhang et al., 2020; Arroyo-García et al., 2021). We add to this literature by demonstrating that KA-driven gamma oscillations in brain slices from *5xFAD* mice reveal deficits at an earlier stage of disease progression, and of greater impact than cholinergically-driven gamma oscillations. As mentioned previously, the triggering mechanisms behind KA- and Cch-driven gamma oscillations are distinct involving unique receptors with differing expression profiles within the hippocampus (Buhl et al., 1998; Fisahn et al., 2002, 2004; Fisahn, 2005). It is also possible that the two approaches engage distinct populations of cells, which are differently affected by evolving AD pathology. This has previously been proposed in work comparing cholinergic, and metabotropic glutamatergic oscillations (Pálhalmi et al., 2004). Additionally, gamma oscillations induced by KA were significantly higher power than those induced by Cch. Thus, our findings could reflect a requisite minimum threshold level of cellular participation to reveal deficits in pharmacologically induced gamma oscillations associated with AD pathology. If only certain subpopulations of cells are affected in AD, then perhaps the gamma deficit phenotype does not reveal itself unless the network is driven particularly hard, as happens with the network activity induced by KA.

*5xFAD* mice also yielded a lower percentage of slices with detectable CA1 gamma oscillations, and impairments in synchrony between CA3 and CA1, manifested as reduced peak cross-correlations (Figures 3, 5). Despite this, in slices with coupled CA3-CA1 gamma oscillations, there was no difference in the phase lag between regions, suggesting a deficit in CA1 recruitment independent of severe Schaffer collateral impairment. The CA1 deficits instead could reflect weaker population drive or coordination of CA1, through post-synaptic deficits, or CA1 circuit cell-specific excitability impairments. A prior study investigating synaptic function in behaving *5xFAD* mice found impaired inhibitory interneuronal connections onto CA1 pyramidal cells and disrupted sharp-wave ripples (Prince et al., 2021). A different study similarly found altered SWRs and PV basket cell (PVBC) activity in the CA1 of 3 month *5xFAD* brain slices, with reduced excitatory input to PVBCs during SWRs, and reduced SWR-associated firing of PVBCs (Caccavano et al., 2020). Although the two studies reported contrasting findings with regards to SWR frequency and amplitude, they both implicated PVIN dysfunction in their findings. Moreover, J20-Tg-AD mice exhibit subicular/CA1 PVIN dysfunction with reduced maximal firing and impaired input/output functions, that alter fast and slow gamma-theta cross-frequency coupling (Mondragón-Rodríguez et al., 2018). These studies support the possibility that reduced CA1 recruitment during gamma oscillations is caused by impaired CA1 PVIN activity, and/or CA3-CA1 communication deficits.

### Hyperexcitability in Alzheimer’s Disease

Many studies have reported hyperexcitability and altered inhibition/excitation ratios in the brains of AD patients and mouse models (Bakker et al., 2012; Sanchez et al., 2012; Verret et al., 2012; Palop and Mucke, 2016; Styr and Slutsky, 2018; Ciccone et al., 2019). As KA acts to increase neuronal excitability and is commonly used experimentally to induce ictal activity (Lévesque and Avoli, 2013; Falcón-Moya et al., 2018), it was expected that some slices would exhibit ictal-like activity (Figure 7). However, there was no increased tendency towards ictal development in the *5xFAD* tissue. We did find that PVINs in aged tissue fired with higher frequency during KA compared to younger tissue and continued firing at higher frequencies following removal of KA (Figure 6B). This difference was only significant between young and old *5xFAD* PVINs during the KA washout. Although a similar trend was seen in the control tissue, this may reflect enhanced PVIN excitability in older *5xFAD* slices.

### PVIN Dysfunction in Alzheimer’s Disease

PVINs are known to be tightly phase-locked to gamma frequency oscillations (Buzśaki and Wang, 2012). We observed PVIN phase-locking to the rising phase of the gamma cycle, in agreement with prior studies (Varga et al., 2014; Andrade-Talavera et al., 2020; Caccavano et al., 2020; Chung et al., 2020; Arroyo-García et al., 2021). Disrupted PVIN phase-locking in different models of AD has been proposed to underly impaired gamma oscillations. Application of Aβ_42_ peptide to acute brain slices during kainate-induced gamma oscillations promoted increased PVIN firing and interfered with PVIN-gamma phase locking (Andrade-Talavera et al., 2020). Similarly, the application of Aβ_1–42_ oligomers to brain slices showed both impaired inhibition from PVIN-PC synapses, with lower IPSCs and increased paired pulse depression; and impaired excitatory drive onto PVINs, with reduced amplitude PVIN-recorded EPSCs (Chung et al., 2020; Park et al., 2020). However, we did not see any difference in the phase or degree of phase-locking, indicating unique cellular mechanisms for different AD models evaluated in rodent brain slices. Direct, acute application of purified Aβ_42_, even at physiological concentrations, could have a stronger effect on slice network function compared to modest deficits observed in slices from *5xFAD* mice with unknown and variable levels of Aβ. Slices from *5xFAD* mice also had a greater opportunity to undergo compensatory, homeostatic circuit reorganisation to cope with disease progression, perhaps blunting the gamma deficits compared to acute Aβ_42_ challenge. However, another recent study reported that APP^NL-G-F^ mice exhibited impaired phase-gamma coupling of fast-spiking interneurons with reductions in phase modulation, but not phase angle, as early as 2 months prior to plaque formation, which could indicate a difference between AD mouse models which do, or do not, overexpress hAPP (Saito et al., 2014; Arroyo-García et al., 2021). Finally, several studies have investigated the contribution of different interneuron subtypes toward gamma frequency oscillations such as CGE-derived trilaminar interneurons, which are strongly phase-locked to gamma oscillations in CA1 (Craig and McBain, 2015); and somatostatin-positive interneurons, which have been implicated in the generation of low-frequency gamma oscillations in the visual cortex *in vivo* and hippocampus *in vitro* (Chen et al., 2017; Veit et al., 2017; Antonoudiou et al., 2020). KA- and Cch- application could differentially recruit these interneuron subtypes, based on their receptor expression profiles, and thus AD-associated deficits in other interneuronal populations could be contributing to the KA-specific gamma deficit we describe.

### Cell Loss in Alzheimer’s Disease

The *5xFAD* model is characterised by its early onset pathology, with regards to plaque onset and behavioural deficits (Oakley et al., 2006). As gamma power will be affected by the number of participating cells, neuronal loss within the hippocampus could be the cause of reduced gamma power. Although we did not measure overall cell density within different hippocampal subfields, *5xFAD* mice reportedly have significant cell loss in the deep cortical cell layers and subiculum by 9–12 months (Jawhar et al., 2012; Eimer and Vassar, 2013); and cell loss in the hippocampus and cortex by 8–9 months, with synapse loss as early as 4 months in the neocortex (Kang et al., 2021). In particular, PVIN loss could impair the rhythmicity of, and/or the ability of the network to generate gamma oscillatory activity. However, reports of PVIN loss in AD are inconclusive. There are reports of significant PVIN loss in patients and mouse models of AD (Brady and Mufson, 1997; Mahar et al., 2017; Crapser et al., 2020; Giesers and Wirths, 2020; Hongo et al., 2020), whereas no GABAergic cell loss was found in 2, 8 or 18 month APP-J20 AD mice (Rubio et al., 2012). There are even reports of increased PV immunoreactivity in 3-month APPPS1 mice (Hollnagel et al., 2019). We examined the hippocampal PVIN density in brain slices from 3 to 4-month and 12+-month-old *5xFAD* mice and found a significant decrease in the total number of PVINs in old *5xFAD* tissue (Figure 8G). While this may in part explain the impaired power and rhythmicity observed, it was striking that no deficits were observed for Cch-induced oscillations and that PVIN function appeared preserved in *5xFAD* mice.

### PVINs and Perineuronal Nets (PNNs) in Alzheimer’s Disease

Perineuronal nets (PNNs) are extracellular matrix structures which form during development and signal the end of the critical period for synaptic plasticity (Sorg et al., 2016). They are thought to be involved in determining levels of plasticity and in stabilising and securing synaptic connections. They have been shown to preferentially surround PVINs (Härtig et al., 1999); and there is evidence for their role in memory impairment in AD (Sorg et al., 2016; Wen et al., 2018). Due to their role in protecting against oxidative stress, some studies argue that impaired PNNs leave cells more susceptible to damage and cell death (Morawski et al., 2004). On the other hand, digesting PNNs with chondroitinase ABC may reopen plasticity, to permit structural remodelling such as axonal sprouting to bypass damaged synapses in AD (Romberg et al., 2013; Yang et al., 2015). There is also evidence for PNN disruption altering the activity of PVINs and PVIN-dependent forms of network activity such as sharp wave ripples and gamma frequency oscillations (Favuzzi et al., 2017; Lensjø et al., 2017; Sun et al., 2018).

The literature is divided on whether or not AD affects PNNs (Morawski et al., 2010; Cattaud et al., 2018; Crapser et al., 2020). Our results show a reduction in total PNNs in older *5xFAD* tissue. However, further work will be necessary to determine whether the loss in PNNs is upstream or downstream of PVIN loss.

### Possible Contribution of Metabolic Stress Towards Impaired Network Activity in AD

Another possible explanation for the gamma deficits uncovered during larger power, KA-driven activity, could be the role of cellular metabolism. This is of particular relevance, considering the strong association between increased metabolic stress and Alzheimer’s disease pathophysiology (de Felice and Lourenco, 2015; Chan et al., 2016). It has been well documented that oxygen consumption and expression of mitochondrial proteins increase with gamma oscillatory activity in the hippocampal slices in an activity-dependent manner (Kann et al., 2011; Chan et al., 2016). Metabolic stress has also been shown to reduce gamma oscillations *in vitro*, particularly in the ventral hippocampus, whilst leaving gamma frequency unchanged, similar to our results (Steullet et al., 2010; Elzoheiry et al., 2020). It is, therefore, possible that deficits in *5xFAD* network activity are only present when driven by kainate as these oscillations are larger power and consequently more metabolically demanding. In this case, metabolic dysfunction within the network would be exacerbated by the intensity of the network activity.

Although one might expect the impact of metabolic stress to manifest primarily in PVINs, due to their energy-demanding physiology, we see no change in PVIN firing properties in brain slices from *5xFAD* mice. This could be attributed to specific PVIN properties intended to manage this energy requirement, such as higher densities of mitochondria with greater cytochrome-c content, increased expression of the mitochondrial transcriptional co-activator PGC-1α, and fast-gating K_V_3 channels (Tansey et al., 2002; Cowell et al., 2007; Kann et al., 2014; Kann, 2016). Additionally, recruitment and synchronisation of cell ensembles during cholinergically-induced gamma oscillations were compromised by rotenone-mediated inhibition of complex 1, whereas individual cell activity was unaffected (Elzoheiry et al., 2020). This suggests that mild metabolic stress, whilst not impacting individual cell activity patterns, could impair the regulation of neuronal ensembles in network gamma activity, possibly through subtle alterations in synaptic function. Indeed, as previously mentioned, the application of Aβ_1–42_ oligomers to hippocampal slices disrupted synaptic transmission at reciprocal PC-PVIN synapses and impaired network oscillations (Kurudenkandy et al., 2014; Chung et al., 2020; Park et al., 2020).

In line with these recent findings, there is interest in exploring antioxidant treatment for AD pathophysiology, to target metabolic stress (Kapogiannis and Mattson, 2011; Hongo et al., 2020; Yan et al., 2020). Likewise, there has also been a lot of focus on developing therapies to boost gamma oscillatory function in order to reduce AD pathology and improve cognitive function (Iaccarino et al., 2016; Lee et al., 2018; Adaikkan et al., 2019; Etter et al., 2019; Jones et al., 2019; Martorell et al., 2019; Strüber and Herrmann, 2020; Zhang et al., 2020). However, it may also be useful to consider issues such as metabolic stress, impaired synaptic transmission, and disrupted gamma frequency activity as overlapping pathologies each with the ability to influence one another (Kapogiannis and Mattson, 2011). In this way, potential combinatorial approaches towards AD treatment may be developed.

## Conclusion

We have described here that KA-driven network activity in the *5xFAD* mouse is impaired; whilst Cch-driven activity is relatively spared in the model, despite extensive plaque burden and loss of PVINs. It has already been acknowledged that plaque burden does not correlate well with the level of cognitive decline in AD (Hardy and Selkoe, 2002). Our work suggests that plaque-levels and even cell loss are not enough to fully predict the extent of network-level deficits in AD, and that the network is strikingly resilient to these pathological changes. In conclusion, this highlights the need for a more nuanced approach towards thinking of Alzheimer’s disease pathology in order to improve understanding of disease aetiology, diagnosis, and treatment options.

## Data Availability Statement

The raw data supporting the conclusions of this article will be made available by the authors, without undue reservation.

## Ethics Statement

The animal study was reviewed and approved by National Institutes of Health (ASP# 17-045).

## Author Contributions

CM and KP conceived and designed research. CM-G, KP, and AC performed electrophysiology experiments. CM-G and AC analysed electrophysiology data. CM-G, ML, KB, and NB performed/contributed to immunohistochemistry experiments or analysis. DA maintained and managed transgenic mouse colonies. CM, KP, AC, and CM-G interpreted results of experiments. AC and AT provided code for electrophysiology analysis. CM-G prepared figures and drafted manuscript. KP, AC, AT, and CM edited and revised manuscript. All authors approved final version of manuscript. All authors contributed to the article and approved the submitted version.

## Conflict of Interest

The authors declare that the research was conducted in the absence of any commercial or financial relationships that could be construed as a potential conflict of interest.

## Publisher’s Note

All claims expressed in this article are solely those of the authors and do not necessarily represent those of their affiliated organizations, or those of the publisher, the editors and the reviewers. Any product that may be evaluated in this article, or claim that may be made by its manufacturer, is not guaranteed or endorsed by the publisher.
